# Supplementary material for: TmSpz6 Is Essential for Regulating the Immune Response to Escherichia coli and Staphylococcus aureus Infection in Tenebrio molitor
Source: Insects. 2020 Feb 5;11(2):105. doi: 10.3390/insects11020105 (PMC7074004; doi:10.3390/insects11020105)
Supplement: Supplementary file 1 [file insects-11-00105-s001.pdf]

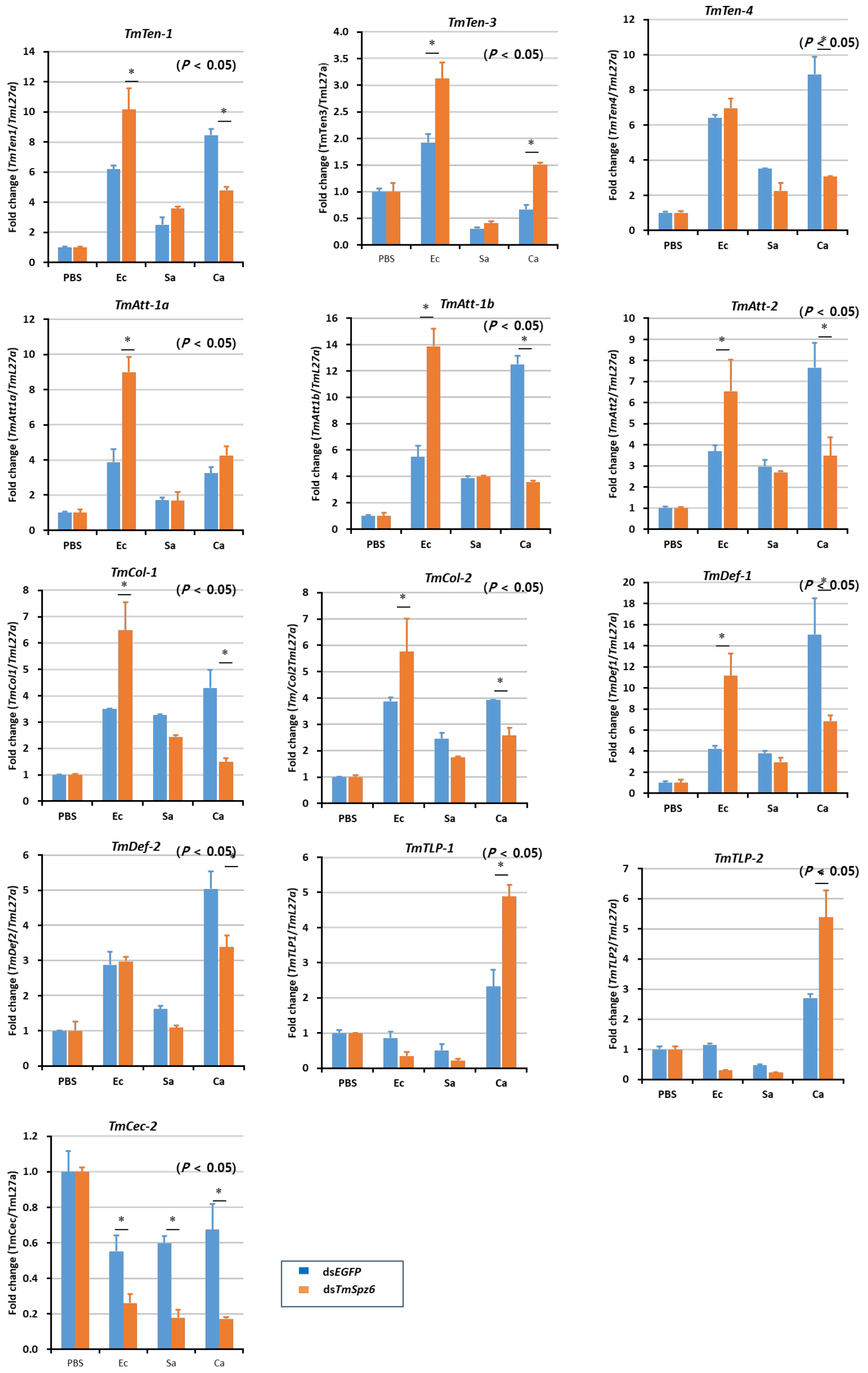

Figure S1. Antimicrobial peptide (AMP) gene induction patterns in the hemocytes of *TmSpz6*-silenced larvae. AMP gene expression levels in the hemocytes of *TmSpz6*-knockdown *T. molitor* larvae were assessed after injection with *E. coli* (Ec), *S. aureus* (Sa), or *C. albicans* (Ca). PBS was injected as a control at 5 days post-*TmSpz4* silencing. At 24 h post-microbial challenge, the expression levels of the following AMP genes were measured by qRT-PCR: *TmTen-1*, *TmTen-2*, *TmTen-3*, *TmTen-4*, *TmAtt-1a*, *TmAtt-1b*, *TmAtt-2*, *TmDef-1*), *TmDef-2*, *TmCol-1*, *TmCol-2*, *TmCec-2*, *TmTLP-1*, and *TmTLP-2*. ds*EGFP* was injected as a negative control, and *TmL27a* expression was assessed as an internal control. All experiments were performed in triplicate. Asterisks indicate significant differences between ds*TmSpz6*- and ds*EGFP*-treated groups when compared by Student's t-test ($p \leq 0.05$).

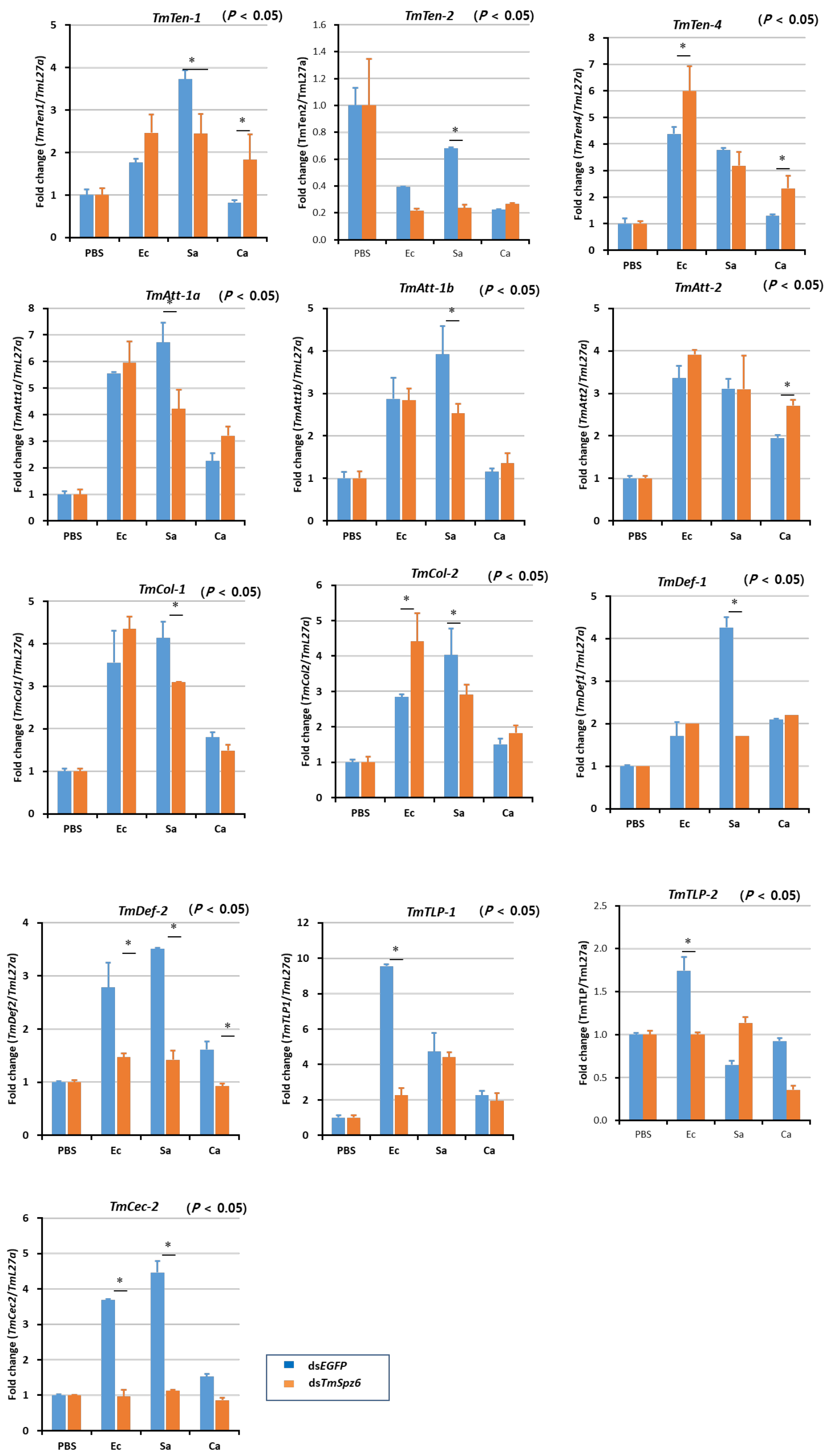

Figure S2. Antimicrobial peptide (AMP) gene induction patterns in the fat body of *TmSpz6*-silenced larvae. AMP gene expression levels in the fat body of *TmSpz6*-knockdown *T. molitor* larvae were assessed after injection with *E. coli* (Ec), *S. aureus* (Sa), or *C. albicans* (Ca). PBS was injected as a control at 5 days post-*TmSpz4* silencing. At 24 h post-microbial challenge, the expression levels of the following AMP genes was measured by qRT-PCR: *TmTen-1*, *TmTen-2*, *TmTen-3*, *TmTen-4*, *TmAtt-1a*, *TmAtt-1b*, *TmAtt-2*, *TmDef-1*), *TmDef-2*, *TmCol-1*, *TmCol-2*, *TmCec-2*, *TmTLP-1*, and *TmTLP-2*. ds*EGFP* was injected as a negative control, and *TmL27a* expression was assessed as an internal control. All experiments were performed in triplicate. Asterisks indicate significant differences between ds*TmSpz6*- and ds*EGFP*-treated groups when compared by Student's t-test ($p \leq 0.05$).

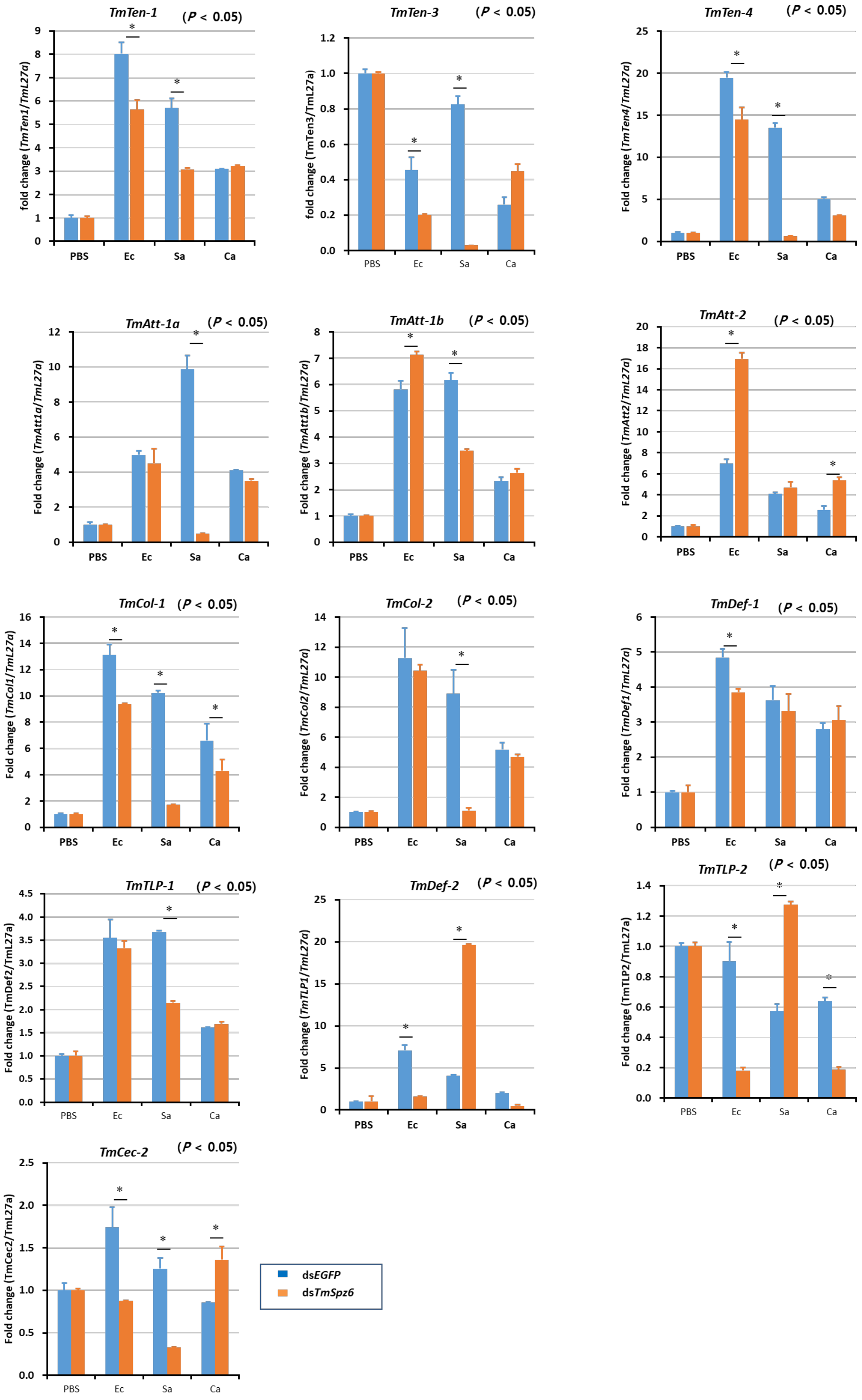

Figure S3. Antimicrobial peptide (AMP) gene induction patterns in the gut of *TmSpz6*-silenced larvae. AMP gene expression levels in the gut of *TmSpz6*-knockdown *T. molitor* larvae were measured after injecting *E. coli* (Ec), *S. aureus* (Sa), or *C. albicans* (Ca). PBS was injected as a control at 5 days post-*TmSpz4* silencing. At 24 h post-microbial challenge, the expression levels of several AMP genes, including *TmTen-1*, *TmTen-2*, *TmTen-3*, *TmTen-4*, *TmAtt-1a*, *TmAtt-1b*, *TmAtt-2*, *TmDef-1*, *TmDef-2*, *TmCol-1*, *TmCol-2*, *TmCec-2*, *TmTLP-1*, and *TmTLP-2*, were measured by qRT-PCR. ds*EGFP* was injected as a negative control for ds*TmSpz6*, and *TmL27a* was measured as an internal control. All experiments were performed in triplicate. Asterisks indicate significant differences between ds*TmSpz6*- and ds*TmEGFP*-treated groups when compared by Student's t-test ($p \leq 0.05$).
